# House-plants as Sanitary Agents

**Published:** 1887-03

**Authors:** 


HOUSE-PLANTS AS SANITARY AGENTS; OR, THE RELATION OF GROWING VEGETATION TO HEALTH AND DISEASE. *By* J. M. ANDERS, M.D., Ph.D. *Pp. 334. Philadelphia:* J. P. LIPPINCOTT COMPANY. *1886.*

This interesting work contains a great deal of information on an important subject. The author has published several papers during the last few years on the chemical activity of growing plants, and the results given in these with much new matter, and abstracts of the work of others, is here presented in book form. The value of house-plants as agents in the purification of air has been discussed pro and con; the numerous observations of Dr. Anders seem to show conclusively that the good they accomplish is generally underestimated.

